# Weakly supervised segmentation models as explainable radiological classifiers for lung tumour detection on CT images

**DOI:** 10.1186/s13244-023-01542-2

**Published:** 2023-11-19

**Authors:** Robert O’Shea, Thubeena Manickavasagar, Carolyn Horst, Daniel Hughes, James Cusack, Sophia Tsoka, Gary Cook, Vicky Goh

**Affiliations:** 1 Department of Cancer Imaging, King’s College London, London, UK; 2 Department of Radiology, Guy’s and St Thomas’ NHS Foundation Trust, London, UK; 3 Department of Radiology, Liverpool University Hospitals NHS Foundation Trust, Liverpool, UK; 4 Department of Natural and Mathematical Sciences, King’s College London, London, UK; 5 King’s College London & Guy’s and St Thomas’ PET Centre, Guy’s and St Thomas’ NHS Foundation Trust, London, UK

**Keywords:** Explainable artificial intelligence, Model interpretation, Weakly supervised learning, Lung neoplasms, Tumour segmentation

## Abstract

**Purpose:**

Interpretability is essential for reliable convolutional neural network (CNN) image classifiers in radiological applications. We describe a weakly supervised segmentation model that learns to delineate the target object, trained with only image-level labels (“image contains object” or “image does not contain object”), presenting a different approach towards explainable object detectors for radiological imaging tasks.

**Methods:**

A weakly supervised Unet architecture (WSUnet) was trained to learn lung tumour segmentation from image-level labelled data. WSUnet generates voxel probability maps with a Unet and then constructs an image-level prediction by global max-pooling, thereby facilitating image-level training. WSUnet’s voxel-level predictions were compared to traditional model interpretation techniques (class activation mapping, integrated gradients and occlusion sensitivity) in CT data from three institutions (training/validation: *n* = 412; testing: *n* = 142). Methods were compared using voxel-level discrimination metrics and clinical value was assessed with a clinician preference survey on data from external institutions.

**Results:**

Despite the absence of voxel-level labels in training, WSUnet’s voxel-level predictions localised tumours precisely in both validation (precision: 0.77, 95% CI: [0.76–0.80]; dice: 0.43, 95% CI: [0.39–0.46]), and external testing (precision: 0.78, 95% CI: [0.76–0.81]; dice: 0.33, 95% CI: [0.32–0.35]). WSUnet’s voxel-level discrimination outperformed the best comparator in validation (area under precision recall curve (AUPR): 0.55, 95% CI: [0.49–0.56] vs. 0.23, 95% CI: [0.21–0.25]) and testing (AUPR: 0.40, 95% CI: [0.38–0.41] vs. 0.36, 95% CI: [0.34–0.37]). Clinicians preferred WSUnet predictions in most instances (clinician preference rate: 0.72 95% CI: [0.68–0.77]).

**Conclusion:**

Weakly supervised segmentation is a viable approach by which explainable object detection models may be developed for medical imaging.

**Critical relevance statement:**

WSUnet learns to segment images at voxel level, training only with image-level labels. A Unet backbone first generates a voxel-level probability map and then extracts the maximum voxel prediction as the image-level prediction. Thus, training uses only image-level annotations, reducing human workload. WSUnet’s voxel-level predictions provide a causally verifiable explanation for its image-level prediction, improving interpretability.

**Key points:**

• Explainability and interpretability are essential for reliable medical image classifiers.

• This study applies weakly supervised segmentation to generate explainable image classifiers.

• The weakly supervised Unet inherently explains its image-level predictions at voxel level.

**Graphical Abstract:**

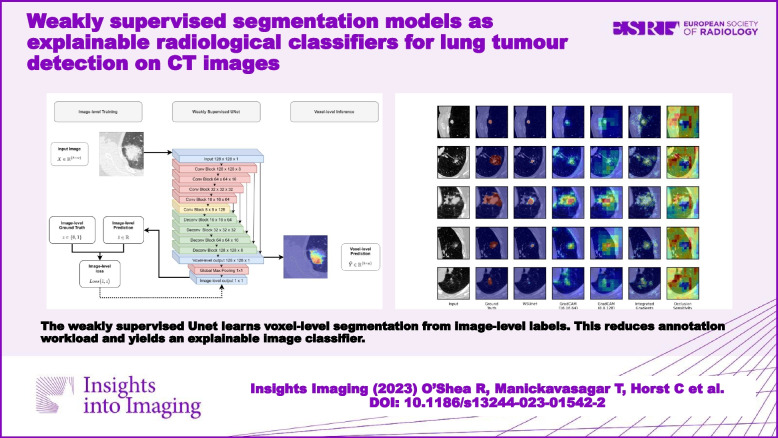

**Supplementary Information:**

The online version contains supplementary material available at 10.1186/s13244-023-01542-2.

## Introduction

Explainability is a well-known limitation of convolutional neural network (CNN) image classifiers, whose “black-box” nature presents various clinical issues [1–3]. Traditionally, radiologists justify diagnoses with corresponding image findings, providing evidence which is independently verifiable. In contrast, CNN decisions are not explicitly justified. Consequently, it can be difficult to verify that a model has made an appropriate prediction, using relevant image features.

Confounding, where images are classified based on spurious features, poses risks of misclassification, discrimination, and vulnerability to adversarial attacks in CNN models [1, 3–5]. For example, Badgeley demonstrated that a model predicting fracture in hip radiographs depended significantly upon patient characteristics and image acquisition parameters, failing to discriminate fractures from normal radiographs when these factors were controlled [5]. The absence of justification for CNN decisions complicates interpretation by clinicians and patients alike, compromising responsibility, communication and capacity to consent [2, 3]. For these reasons, explainability is a central component of the European Commission’s Assessment List for Trustworthy Artificial Intelligence [6], a key guideline for the prospective regulation of artificial intelligence development in high-risk applications such as healthcare.

As standard CNN decision functions are not easily invertible [7], voxels’ contributions to the image-level predictions are unavailable. This limitation motivated the development of saliency mapping techniques to reverse engineer CNN decisions and extract the regions of interest. Class activation mapping [8–10], occlusion sensitivity [11] and gradient integration are commonly employed approaches [12].

Conceptually, the problem of explainable image classification is closely related to that of weakly supervised semantic segmentation, where voxel-level classification labels are modelled from image level labelled at the image level. Several weakly supervised segmentation methods utilise class activation maps to generate an initial voxel-level probability map, which serves as a pseudo-label for subsequent model training [13–15]. Oquab et al. introduced voxel-pooling to construct image-level predictions from voxel-level predictions, thereby facilitating segmentation modelling with only image-level supervision [16].

Global pooling allows voxel-level predictions to be aggregated into image-level labels, under the pretext that positive voxels imply positive images [16]. Global max-pooling has yielded precise object localisation in general-purpose imaging tasks [17], presenting an avenue for application to explainable medical image classification. However, biomedical applications present several challenges for weakly supervised segmentation, including class imbalance, low contrast between positive regions and background, and variability in the appearance of positive regions [18, 19].

This study presents a weakly supervised Unet architecture (WSUnet) which learns to localise lung tumours through comparison of positive and negative images, thereby yielding an interpretable lung tumour detection model. WSUnet’s voxel-level segmentations are compared to commonly used model interpretation methods for tumour delineation.

## Materials and methods

This study was performed in accordance with the Checklist for Artificial Intelligence in Medical Imaging (CLAIM) guidelines [20]. The CLAIM checklist is provided in Supplementary Table 1.

### Model development

**Fig. 1:**
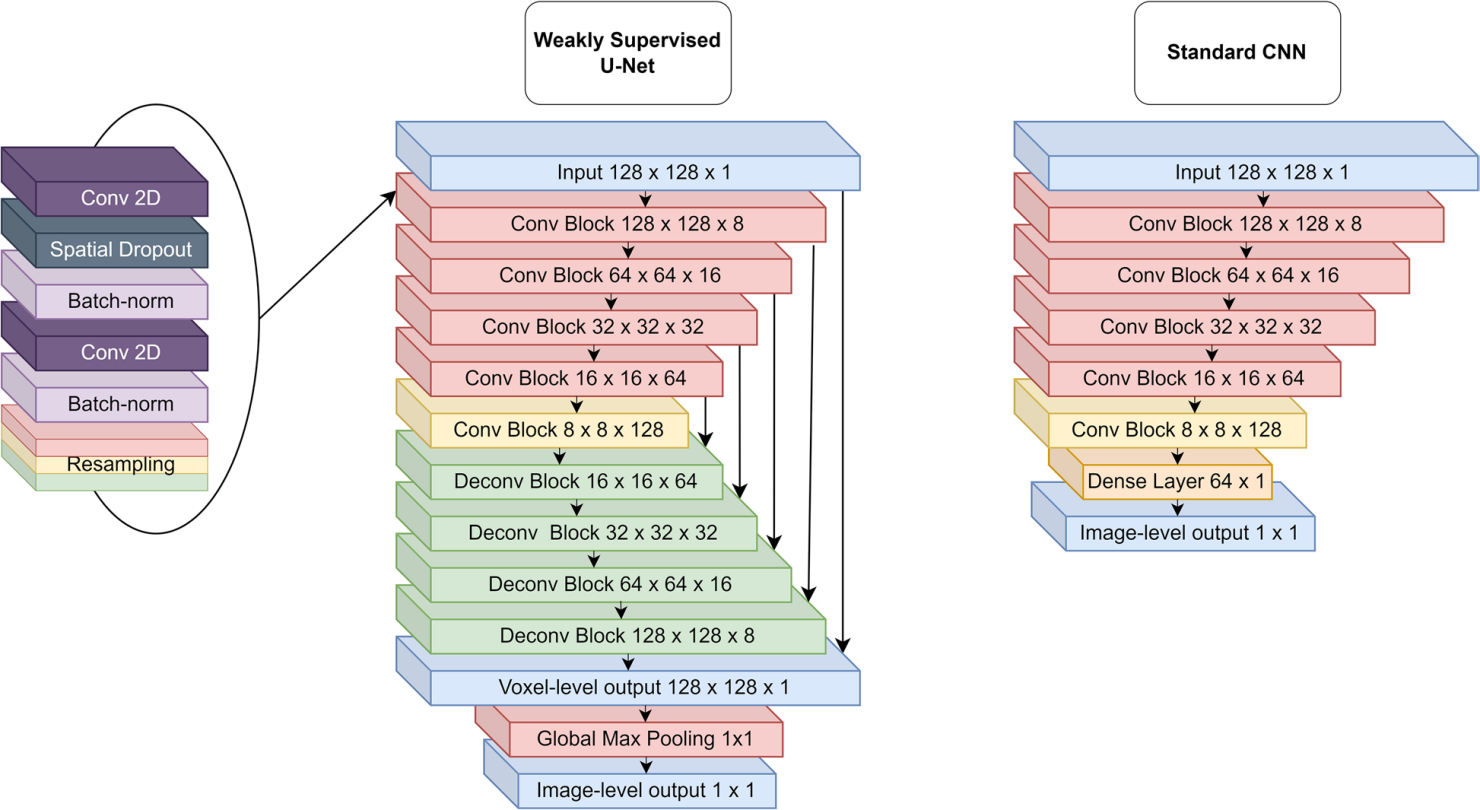
Weakly supervised Unet and standard CNN architectures. Blue layers represent inputs and outputs. Red, green and yellow layers represent downsampling, upsampling, and non-resampling convolutional blocks, respectively. Purple, charcoal and lilac layers represent convolutional, dropout and normalisation layers, respectively. Orange layers represent dense layers

A weakly supervised Unet (WSUnet) was constructed by appending a global max-pooling layer to the output of a Unet with five convolutional and four deconvolutional blocks (487 k trainable parameters). Comparator models were generated using a “standard” CNN pyramid (sCNN) with equivalent architecture to the UNet encoder (1344 k trainable parameters) and a DenseNet-121 architecture [21]. WSUnet and sCNN model architectures are illustrated in Fig. 1. Model weights were randomly initialised. Rectified linear activation was applied to hidden layers and sigmoid activation to the output layers. Spatial dropout was applied with a rate of 0.1. The models were optimised using the Adam optimiser with a learning rate of 0.001 using binary cross-entropy loss at the image level. Training and validation partitions were created from a 5-fold patient-disjoint partition of the Aerts dataset. Training images were randomly augmented with horizontal flips, vertical flips and rotations by 90, 180 and 270°. In each fold, training continued until validation loss plateaued for 5 epochs. To examine the progression and stability of WSUnet’s voxel-level performance over time, a separate run was conducted in which WSUnet models were fitted in cross-validated training over 25 epochs, with voxel-level metrics measured in validation data after each epoch. Model training was performed with Tensorflow v2.4.0, Keras v2.2.4 and keras-unet version v0.1.2 [22–24]. Four model interpretation methods were applied to generate voxel-level predictions from Densenet and sCNN models, using code extracted and modified from tf-explain v0.3.1 [25]. GradCAM heatmaps were extracted from the seventh convolutional layer (GradCAM (16, 16, 64)) and the ninth convolutional layer (GradCAM (8, 8, 128)). Nearest-neighbour interpolation was applied to scale the grad-cam outputs to the input dimension ($128\times 128$). Occlusion sensitivity was applied with an occlusion width of 10 voxels. Integrated gradients were measured with 10 whitening steps.

All computation was performed on a desktop operating Windows 11 with 32 GB random access memory, an Intel Xeon Silver 4114 central processing unit and an Nvidia Quadro P2000 graphics card. All analysis was performed in the python language v3.7. All code required to reproduce the results of this analysis is provided at https://github.com/robertoshea/wsss/.

### Model testing

**Fig. 2:**
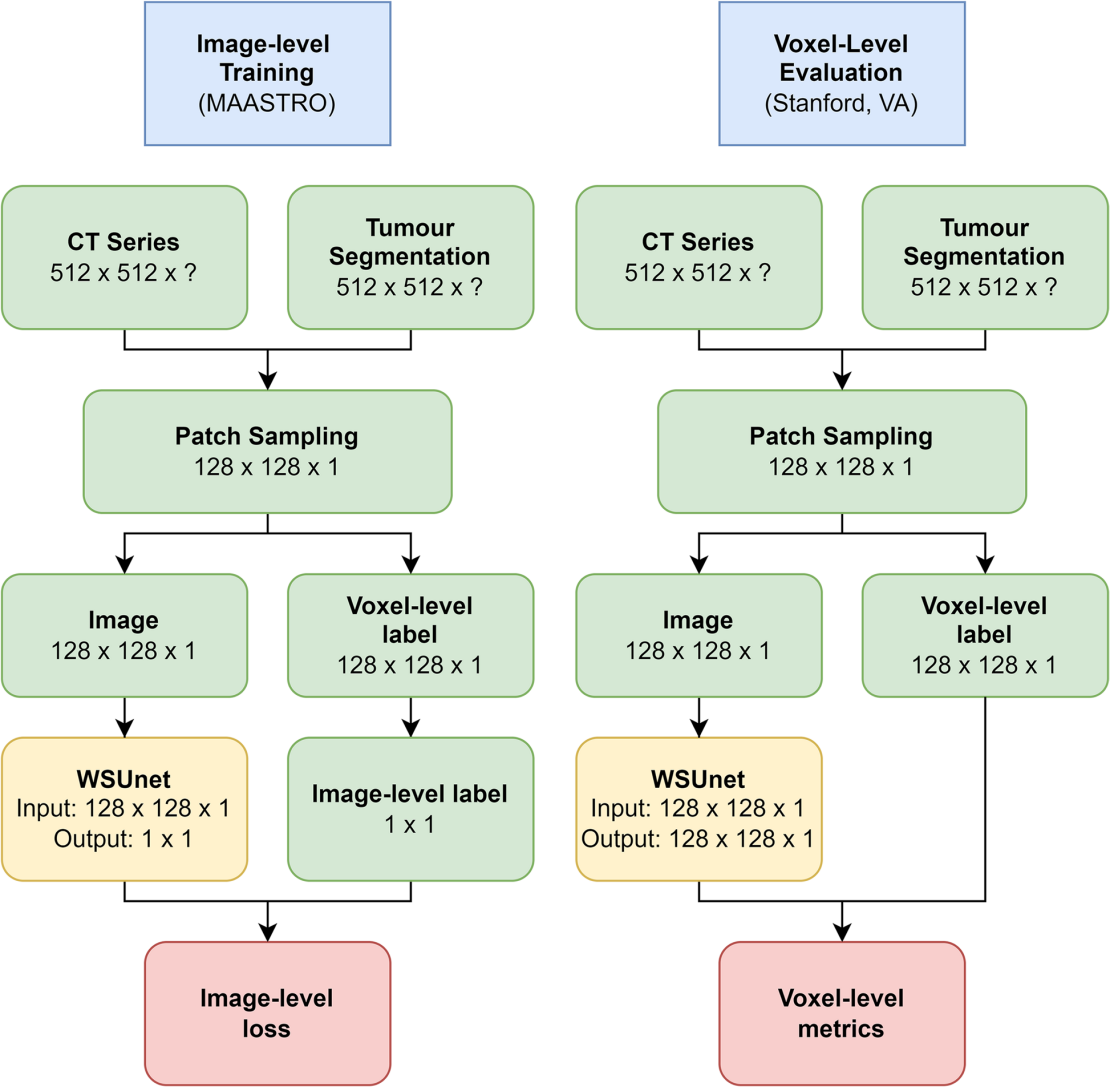
Model training and evaluation schema. Image patches were sampled in the MAASTRO dataset, and labelled at image level according to whether the image contained any tumour-positive voxels. Model training utilised the image-level labels only; the model was not provided with any information on tumour location. The model evaluation used both image-level labels and voxel-level ground truth

Test performance was evaluated in the Stanford/VA dataset. Figure 2 provides a schema of model training and testing. Voxel-level NSCLC discrimination was evaluated using area under the precision-recall curve (AUPR). As WSUnet returns class probability estimates, calibration was also computed in terms of precision, recall and dice score, discretising by a threshold of 0.5. Calibration was assessed using the expected calibration error metric [26]. As methods other than WSUnet do not yield class probabilities, calibration metrics were not computable. Voxel-level metrics are defined in the Supplementary information. Image-level classification was also evaluated with accuracy, sensitivity, specificity and area under the receiver operating characteristic curve (AUC) metrics. Clinical evaluation of voxel-level outputs was performed by clinicians with subspecialty experience in thoracic cancer imaging, including one staff radiologist (V.G.), two specialist radiology residents (C.H., J.C.) and two specialist oncology residents (T.H., D.H.). One hundred images with tumours present were extracted from the test set, and each voxel-level prediction method was applied. Clinicians were provided with input images, ground truth and a blinded, random ordering of each methods’ voxel-level predictions.

Clinicians selected the method which they considered the most clinically useful in each test instance. Clinician preference rate was calculated as the frequency with which clinicians preferred the method, excluding instances in which they considered all methods uninformative. Clinicians also rated the tumour detection difficulty in each image (1: “tumour obvious”, 2: “tumour difficult to identify”, 3: “tumour not visible in this image”). The clinician preference survey is provided in Supplementary Data 1.

Metric distributions were estimated with 500 nonparametric bootstraps and 95% confidence intervals for all metrics were estimated according to the 2.5th and 97.5th centiles of the bootstrap distribution.

### Experimental datasets

WSUnet was evaluated by application to NSCLC detection and localisation, using data from The Cancer Imaging Archive [27]. Model development was performed with the MAASTRO dataset [28, 29], which contains annotated retrospective CT data from 422 inoperable, pathologically confirmed, stage I-IIIb (non-metastatic tumour limited to lung, adjacent structures and ipsilateral hilar and mediastinal lymph nodes, without malignant effusion) NSCLC patients at Maastricht University Medical Centre. Testing was performed in the Stanford/VA dataset [30, 31], which contains annotated retrospective CT data from 211 early-stage (non-metastatic tumours limited to lung and ipsilateral hilar and mediastinal lymph nodes, or limited to adjacent tissues without lymphatic metastasis) NSCLC patients referred for surgical management at Stanford School of Medicine and the Veterans Association Hospital Palo Alto. Further information on the study data is provided in Supplementary Information. Subjects were excluded if CT images with tumour segmentations were unavailable, or if the gross tumour volume was not clearly identifiable in the annotation region labels. Voxel intensity arrays were extracted from CT DICOM volumes, converted to Hounsfield units and scaled by a factor of 0.001 using the pydicom library v2.1.2 [32]. In each CT volume, axial patches of dimension $128\times 128\times 1$ voxels were sampled. Forty patches were sampled with centrepoints in the tumour volume. 40 patches were sampled from the contralateral lung by mirroring the tumour voxels sagitally and randomly offsetting points by $\pm\ 15$ voxels axially, $\pm\ 75$ voxels coronally and $\pm\ 75$ voxels sagitally. Image patches were labelled positive if they contained any tumour voxel and negative otherwise.

**Fig. 3:**
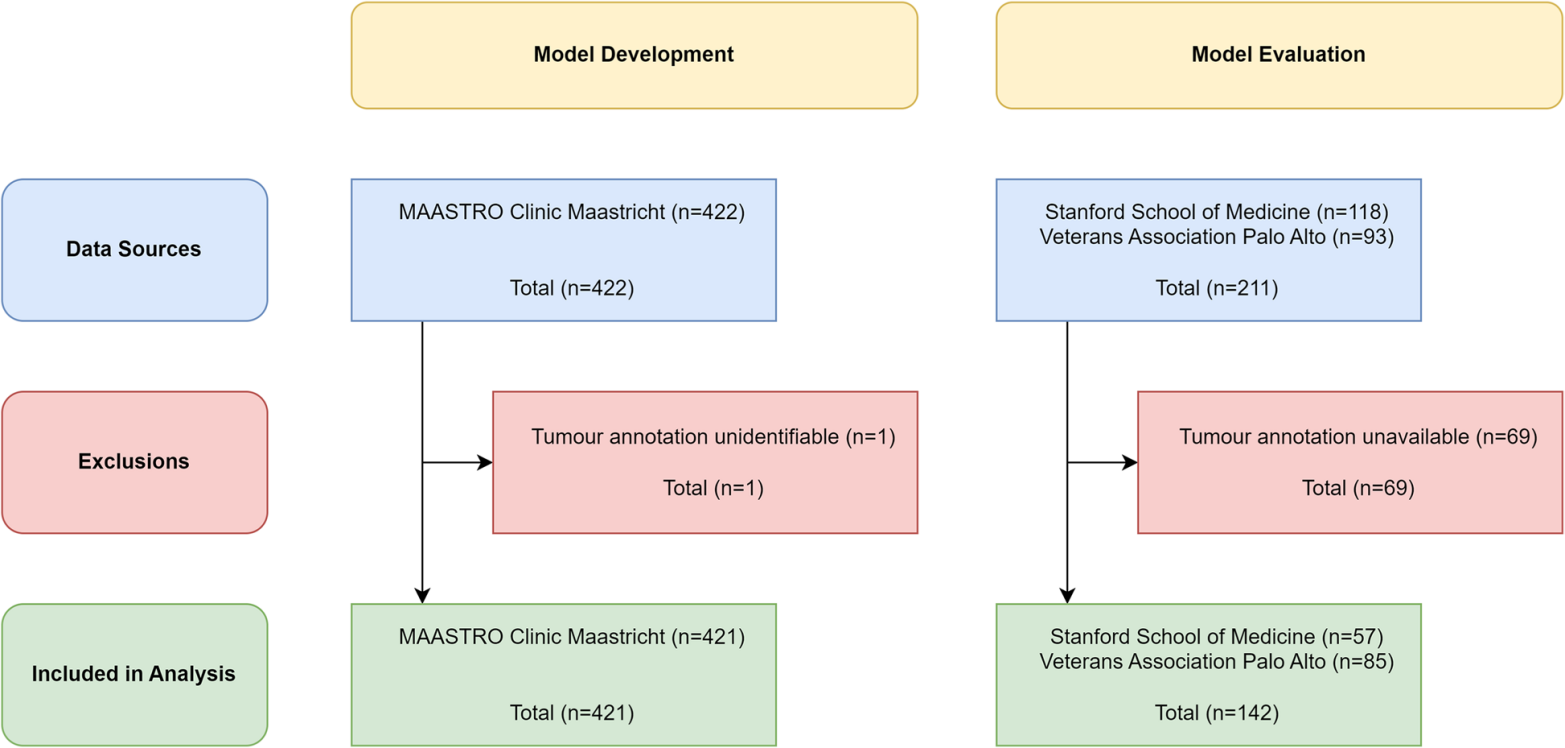
Flowchart of data sources, exclusions and inclusions

One subject was excluded from the MAASTRO dataset as the gross tumour volume label could not be identified definitively, and 69 subjects were excluded from the Stanford/VA dataset due to the absence of segmentation data. Thus, 421 subjects were included for model development and 142 subjects were included for model evaluation. Clinical characteristics of the training and testing cohorts are described in Table 1. Image acquisition parameters are provided in Supplementary Table 2. A flowchart of study participants is provided in Fig. 3.

**Table 1:** Clinical characteristics of the study population. Stage represents the clinical stage in the training data and the pathological stage in test data

**Variable**	**Value**	***N* (train)**	***N* (test)**
Institution	Maastricht University Medical Centre	421	0
	Stanford University Medical School Hospital	0	57
	Veterans Association Palo Alto	0	85
Age (years)			
[Range]	20–40	1	0
	41–60	84	17
	61–80	273	112
	81–100	41	13
Sex	Female	132	36
	Male	289	106
T-stage	1	93	68
	2	155	48
	3	53	16
	4	117	5
	Unknown	3	5
N-stage	0	170	113
	1	23	12
	2	140	17
	3	85	0
	Unknown	3	0
M-stage	0	416	138
	1	1	4
	Unknown	4	0
Histology	Adenocarcinoma	51	111
	Large cell carcinoma	114	0
	Squamous cell carcinoma	151	28
	Other	63	3
Scanner manufacturer	GE Medical Systems	0	116
	Philips	0	2
	Siemens	421	14
	Toshiba	0	1

## Results

### Objective performance metrics

Voxel-level classification (segmentation) performance is provided in Table 2. WSUnet’s voxel-level outputs localised NSCLC regions precisely in both validation (precision: 0.77 [96% CI: 0.75–0.80]; dice: 0.43, 95% CI: [0.39–0.46]) and test instances (precision: 0.78, 95% CI: [0.76–0.81]; dice: 0.33, 95% CI: [0.32–0.35]). However, WSUnet’s voxel-level performance was limited by low recall in test instances (recall: 0.24, 95% CI: [0.22–0.25]), decreasing from validation set recall (recall: 0.33, 95% CI: [0.29–0.36]). WSUnet demonstrated strong discrimination at voxel level in validation (AUPR: 0.55, 95% CI: [0.54–0.55]), significantly outperforming the closest alternative, sCNN GradCAM (16, 16, 64) (AUPR: 0.28, 95% CI: [0.25–0.30]). Although WSUnet achieved the highest test discrimination (AUPR: 0.40, 95% CI: [0.38–0.41]), comparable performance was achieved by sCNN GradCAM (16, 16, 64) (AUPR: 0.36, 95% CI: [0.34–0.37]).

**Table 2:** Voxel-level NSCLC classification performance. Mean values and 95% confidence intervals are provided. Calibration metrics were not computable for GradCAM, integrated gradients and occlusion sensitivity methods. The clinician preference rate was calculated as the frequency with which clinicians preferred the method in 100 test instances, excluding instances in which they considered no model informative. DenseNet predictions were not included in the clinician preference test

**Partition**	**Method**	**Precision**	**Recall**	**Dice**	**ECE**	**AUPR**	**Clinician preference rate**
Validation	WSUnet	0.77 [0.75–0.8]	0.33 [0.29–0.36]	0.43 [0.39–0.46]	0.02 [0.01–0.02]	0.53 [0.49–0.56]	NA
Validation	sCNN GradCAM (16, 16, 64)	NA	NA	NA	NA	0.28 [0.25–0.3]	NA
Validation	sCNN GradCAM (8, 8, 128)	NA	NA	NA	NA	0.27 [0.25–0.29]	NA
Validation	sCNN integrated gradients	NA	NA	NA	NA	0.1 [0.09–0.1]	NA
Validation	sCNN occlusion sensitivity	NA	NA	NA	NA	0.04 [0.03–0.04]	NA
Validation	DenseNet GradCAM (16, 16, 64)	NA	NA	NA	NA	0.19 [0.17–0.21]	NA
Validation	DenseNet GradCAM (8, 8, 128)	NA	NA	NA	NA	0.23 [0.21–0.25]	NA
Validation	DenseNet occlusion sensitivity	NA	NA	NA	NA	0.03 [0.02–0.03]	NA
Test	WSUnet	0.78 [0.76–0.81]	0.24 [0.22–0.25]	0.33 [0.32–0.35]	0.01 [0.01–0.02]	0.4 [0.38–0.41]	0.72 [0.68–0.77]
Test	sCNN GradCAM (16, 16, 64)	NA	NA	NA	NA	0.36 [0.34–0.37]	0.2 [0.16–0.24]
Test	sCNN GradCAM (8, 8, 128)	NA	NA	NA	NA	0.23 [0.21–0.24]	0.05 [0.03–0.08]
Test	sCNN integrated gradients	NA	NA	NA	NA	0.11 [0.1–0.11]	0.01 [0.0–0.03]
Test	sCNN occlusion sensitivity	NA	NA	NA	NA	0.03 [0.03–0.03]	0.0 [0.0–0.01]
Test	DenseNet GradCAM (16, 16, 64)	NA	NA	NA	NA	0.13 [0.12–0.14]	NA
Test	DenseNet GradCAM (8, 8, 128)	NA	NA	NA	NA	0.23 [0.22–0.25]	NA
Test	DenseNet occlusion sensitivity	NA	NA	NA	NA	0.02 [0.02–0.02]	NA

*ECE* Expected calibration error, *AUPR* Area under precision recall curve

Image-level classification results are provided in Table 3. In test instances, WSUnet demonstrated similar image-level classification performance (accuracy: 0.86 [0.85–0.87]; AUC: 0.94 [0.94–0.95]) to sCNN (accuracy: 0.88 [0.87–0.89]; AUC: 0.96 [0.95–0.96]) and DenseNet (accuracy: 0.87 [0.86–0.88]; AUC: 0.94 [0.94–0.95]).

**Table 3:** Image-level NSCLC classification results on test instances. Mean values and 95% confidence intervals are provided

**Partition**	**Method**	**Accuracy**	**Sensitivity**	**Specificity**	**AUC**
Validation	DenseNet	0.86 [0.84–0.88]	0.83 [0.8–0.86]	0.89 [0.86–0.92]	0.94 [0.93–0.96]
Validation	WSUnet	0.87 [0.85–0.89]	0.86 [0.83–0.88]	0.88 [0.86–0.9]	0.95 [0.93–0.96]
Validation	sCNN	0.88 [0.86–0.89]	0.9 [0.87–0.92]	0.85 [0.82–0.88]	0.95 [0.93–0.96]
Test	DenseNet	0.87 [0.86–0.88]	0.86 [0.85–0.87]	0.88 [0.87–0.89]	0.94 [0.94–0.95]
Test	WSUnet	0.86 [0.85–0.87]	0.87 [0.86–0.89]	0.85 [0.84–0.86]	0.94 [0.94–0.95]
Test	sCNN	0.88 [0.87–0.89]	0.84 [0.83–0.85]	0.93 [0.92–0.94]	0.96 [0.95–0.96]

**Fig. 4:**
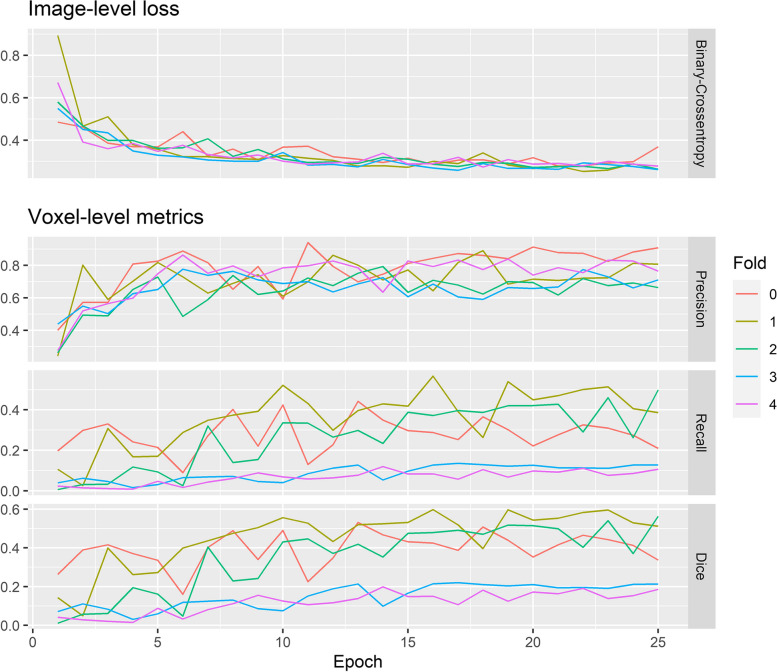
Weakly supervised Unet models’ validation loss and metrics after each epoch of training. Image-level binary cross-entropy was employed for model training. Distinctly coloured curves represent models fitted to different training curves

WSUnet’s validation performance after each training epoch is plotted in Fig. 4. Although models fitted in different training folds demonstrated similar image-level loss, voxel-level performance varied considerably between models. Likewise, whilst image-level loss stabilised after 15 training epochs, voxel-level metrics demonstrated persistent variability from epoch to epoch.

### Clinicians’ performance assessment

Clinicians considered some positive instances moderately difficult to identify, assigning difficulty levels of “Tumour difficult to identify” and “Tumour not visible in this image” to 25.8% and 6% of test images, respectively. In many cases, ground glass changes were the only visible finding. Clinicians strongly preferred WSUnet’s voxel-level outputs to current explainability methods. Excluding instances where no method was considered informative (26%), WSUnet outputs were preferred in 72% of test instances. Clinicians cited “high resolution” and “fine delineation of tumour borders” as reasons motivating their choices. GradCAM (16,16,128) and GradCAM (8,8,64) outputs were preferred in 20% and 5% of test instances, respectively. Integrated gradients and occlusion sensitivity outputs were preferred in fewer than 1% of test instances.

**Fig. 5:**
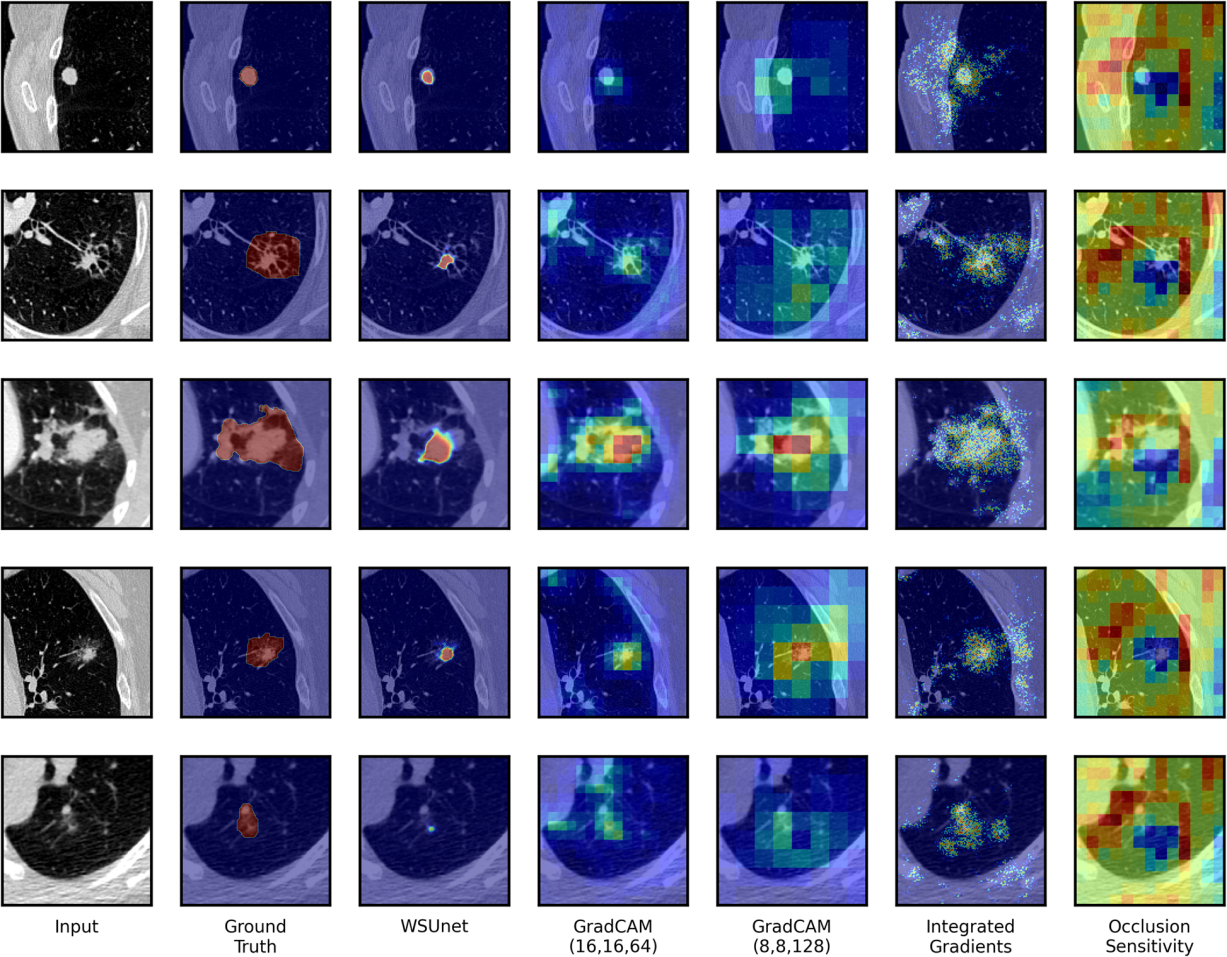
Model explainability heatmaps. The first five positive test instances are shown. Models were trained to detect NSCLC tumours at image level. WSUnet’s heatmap was extracted from the penultimate voxel-level output layer. GradCAM heatmaps were extracted from the seventh (“GradCAM (16, 16, 64)”) and ninth (“GradCAM (8, 8, 128)”) convolutional layers. Nearest-neighbour interpolation was applied to map GradCAM, integrated gradients and occlusion sensitivity heatmap to the input image dimensions. For comparability, methods heatmaps were normalised to the range of minimum and maximum values for the five images

Methods’ voxel-level outputs are provided in Fig. 5. Inspection of WSUnet’s voxel-level output confirms the use of tumoural and peritumoural voxels to generate positive image-level classifications. Although “hot” regions were highly specific to tumour-related areas, several small nodules were missed. Clinical inspection of the WSUnet performance in test instances identified that WSUnet’s low voxel-level recall was partially explained by its specificity for the tumour volume, as the annotated segmentations in the test dataset included peritumoural regions of lung parenchyma. The images in rows 2, 3 and 4 of Fig. 5 provide examples of test instances where WSUnet segmented the tumour volume, but annotation labels included additional peritumoural regions of the lung parenchyma, which were required to achieve high recall performance. GradCAM outputs offered higher sensitivity to small tumours, however, GradCAM (16, 16, 64) marked several ribs as “warm” and the resolution of GradCAM (8, 8, 128) outputs was low. Integrated gradient outputs surrounded the tumour region reliably — however, positive regions were neither continuous nor specific. Occlusion sensitivity outputs were uninformative, differing minimally between inputs.

## Discussion

This study demonstrated that WSUnet learns to localise and segment lung tumours through the comparison of positive and negative images. Thus, the WSUnet architecture serves both as a weakly supervised segmentation method and an explainable image-level classifier.

WSUnet yielded superior voxel-level discrimination to current model interpretation approaches, both by objective and subjective metrics. WSUnet’s voxel-level output identified the voxels motivating the positive image-level prediction, revealing whether the model attended to the tumour or other confounding features. WSUnet offered a distinct advantage of returning predictions in the domain and range of the voxel-level class probabilities, obviating the need for post hoc interpolations and transformations. Thus, WSUnet’s voxel-level output could be interpreted directly as a voxel-probability heatmap.

Although WSUnet’s voxel-level recall did not challenge the state-of-the-art set by fully supervised NSCLC segmentation models trained under full supervision [33], its high precision presents a plausible avenue for object localisation. The low recall performance of WSUnet’s voxel-level predictions provides insight into its reasoning — the model may deduce that the image is positive by finding any tumour region, permitting image-level classification by a small discriminative region of interest. Thus, a positive image-level prediction may be inferred without observing the whole tumour region. Conversely, the whole image must be considered to exclude a tumour. Thus, the model is negatively biased at the voxel level, predisposing it to low recall. This is an important limitation of applying model interpretation methods for weakly supervised segmentation – the model may learn to classify the image using a small discriminative region, leading to undersegmentation. Concurrently, clinicians observed that the voxel-level tumour annotations provided in the Stanford/VA dataset included significant proportions of peritumoural lung parenchyma, which were not segmented by WSUnet, partially explaining apparent under-segmentation performance.

WSUnet’s voxel-level performance was noted to vary between subsequent training epochs, despite stable image-level loss. Furthermore, voxel-level performance appeared to be sensitive to initialisation and early training conditions, as models fitted to different folds demonstrated different voxel-level metrics despite similar image-level performance. These findings demonstrate the limitations of image-level supervision for model selection.

As the saliency map aims to approximate model reasoning, false positive regions typically represent model-misspecification — where the model classified the image on the basis of non-tumoural objects. Conversely, these may represent valid pathobiological associations such as atelectasis. In either case, inspection of the voxel-level predictions improves understanding of the model’s reasoning. However, where the project objective is tumour segmentation, these extra-tumoural pathobiological associations may adversely affect performance by providing an alternative discriminative region.

Although GradCAM predictions localised moderately well to the tumour, their utility was limited by low resolution. Integrated gradient outputs were not locally consistent, such that adjacent voxels typically had dissimilar predictions. Occlusion sensitivity results demonstrated little variance between images. All methods were limited by producing an output which could not be interpreted directly as a voxel-probability map WSUnet is a CNN which returns both an image-level decision and a voxel-level segmentation which motivated the decision. This development facilitates model inspection, debugging, reliability testing, inference and pathobiological discovery. The approach differs from traditional model explainability methods, as the image-level prediction is simply the maximal voxel-level probability. Consequently, voxel-level predictions are interpretable as class probabilities, providing a causally verifiable explanation for the image-level decision. The simple relationship between voxel-level predictions and image-level predictions allows for easy clinical interpretation.

Recent years have seen significant advances towards achieving weakly supervised segmentation for lung CT data. Fruh et al. evaluated class-activation mapping for weakly supervised segmentation of tumours in PET-CT data, attaining a dice score of 0.47 [34]. PET integration may have facilitated the segmentation task, as simple threshold-based segmentation achieved a dice score of 0.29 [34]. Feng et al. applied a global average pooling method to the higher layers of an encoder network to perform weakly supervised segmentation on a lung cancer dataset, achieving high dice scores (0.46–0.54) [35]. The resolution of voxel-level predictions was limited by that of the convolutional layer used for the global average pooling, as interpolation was required to upsample the predictions to the input resolution. Shen et al. proposed a two-stage semi-supervised segmentation approach for lung nodule segmentation, utilising adversarial learning to minimise the discriminability of unsupervised segmentation masks from supervised masks [36]. Laradji et al. proposed consistency-based loss for weakly supervised segmentation modelling of COVID-19-related pneumonitis, where point-level supervision was available [37].

This retrospective study included model evaluation on multi-centre data which was geographically distinct from training data. Training and evaluation datasets included CT images from multiple scanner manufacturers. The study has some limitations. All participants in this study were diagnosed with lung cancer. Consequently, some malignant changes may have been evident in images which did not contain any tumour voxels. In the test data, peritumoural regions were included in tumour segmentation labels, leading to an underestimation of the models’ sensitivity to tumour tissue. Ground truth voxel-level segmentations were employed to identify positive images during the construction of the weakly supervised dataset. The class distribution in this study was approximately balanced at image level and moderately imbalanced at voxel level — the convergence of weak learners may be less reliable in highly imbalanced data. In this study, data was labelled at the level of 128 × 128 axial image patches, whilst clinical applications ideally require tumour localisation in 3D volumes of 512 × 512 image slices. Consequently, further research on the scalability of the method to large, imbalanced datasets is required for clinical utility in typical applications.

In conclusion, this study demonstrated that weakly supervised segmentation is a valid approach by which explainable object detection models may be developed for medical imaging. WSUnet generates a full-resolution voxel-level explanation for its image-level decision, which clinicians found more useful than current model interpretation approaches in application to lung tumour detection. Further research will investigate approaches to improve WSUnet’s voxel-level recall and achieve stable convergence in highly imbalanced data  [21–23, 37].

### Supplementary Information

**Additional file 1.** Supplementary Data.

**Additional file 2:** **Supplementary Table 1.** Checklist for Artificial Intelligence in Medical Imaging (CLAIM)(Mongan et al., 2020) checklist. **Supplementary Table 2.** Acquisition parameters of images used in this analysis.

## Data Availability

All datasets used in this study are publicly available from The Cancer Imaging Archive  [21–23, 37]. All code required to reproduce the findings of this study is provided at github.com/robertoshea/wsss.

## Abbreviations

18F-FDG PET: 18-Fluorine fluorodeoxyglucose positron emission tomography
AUC: Area under receiver operator characteristic curve
AUPR: Area under precision recall curve
CLAIM: Checklist for Artificial Intelligence in Medical Imaging
CNN: Convolutional neural network
CT: Computed tomography
ECE: Expected calibration error
FCN: Fully convolutional neural network
GradCAM: Gradient class activation mapping
NSCLC: Non-small cell lung cancer
PET: Position emission tomography
sCNN: “Standard” CNN (using U-Net encoder architecture)
TNM: Tumour-node-metastasis cancer staging system
WSUnet: Weakly supervised Unet architecture
