# Supplementary material for: Weakly supervised segmentation models as explainable radiological classifiers for lung tumour detection on CT images
Source: Insights Imaging. 2023 Nov 19;14:195. doi: 10.1186/s13244-023-01542-2 (PMC10657919; doi:10.1186/s13244-023-01542-2)
Supplement: Supplementary file 2 — Additional file 2: Supplementary Table 1. Checklist for Artificial Intelligence in Medical Imaging (CLAIM)(Mongan et al., 2020) checklist. Supplementary Table 2. Acquisition parameters of images used in this analysis. [file 13244_2023_1542_MOESM2_ESM.docx]

**Weakly supervised segmentation models as explainable radiological classifiers for lung tumour detection on CT images**

**ELECTRONIC SUPPLEMENTARY MATERIAL**

**Supplementary Table 1.** Checklist for Artificial Intelligence in Medical Imaging (CLAIM)(Mongan et al., 2020) checklist.

| Section / Topic | No. | Item |  |
| --- | --- | --- | --- |
| TITLE / ABSTRACT |  |  |  |
|  | **1** | Identification as a study of AI methodology, specifying the category of technology used (e.g., deep learning) | 1 |
|  | **2** | Structured summary of study design, methods, results, and conclusions | 1 |
| INTRODUCTION |  |  |  |
|  | **3** | Scientific and clinical background, including the intended use and clinical role of the AI approach | **3** |
|  | **4** | Study objectives and hypotheses | **5** |
| METHODS |  |  |  |
| *Study Design* | **5** | Prospective or retrospective study | **6** |
|  | **6** | Study goal, such as model creation, exploratory study, feasibility study, non-inferiority trial | **5** |
| *Data* | **7** | Data sources | **6** |
|  | **8** | Eligibility criteria: how, where, and when potentially eligible participants or studies were identified (e.g., symptoms, results from previous tests, inclusion in registry, patient-care setting, location, dates) | **Supp** |
|  | **9** | Data pre-processing steps | **6** |
|  | **10** | Selection of data subsets, if applicable | **6** |
|  | **11** | Definitions of data elements, with references to Common Data Elements | **N/A** |
|  | **12** | De-identification methods | **Supp** |
|  | **13** | How missing data were handled | **7** |
| *Ground Truth* | **14** | Definition of ground truth reference standard, in sufficient detail to allow replication | **Supp** |
|  | **15** | Rationale for choosing the reference standard (if alternatives exist) | **Supp** |
|  | **16** | Source of ground-truth annotations; qualifications and preparation of annotators | **Supp** |
|  | **17** | Annotation tools | **Supp** |
|  | **18** | Measurement of inter- and intrarater variability; methods to mitigate variability and/or resolve discrepancies | **0** |
| *Data Partitions* | **19** | Intended sample size and how it was determined | **N/A** |
|  | **20** | How data were assigned to partitions; specify proportions | **6** |
|  | **21** | Level at which partitions are disjoint (e.g., image, study, patient, institution) | **6** |
| *Model* | **22** | Detailed description of model, including inputs, outputs, all intermediate layers and connections | **6** |
|  | **23** | Software libraries, frameworks, and packages | **6** |
|  | **24** | Initialization of model parameters (e.g., randomization, transfer learning) | **6** |
| *Training* | **25** | Details of training approach, including data augmentation, hyperparameters, number of models trained | **6** |
|  | **26** | Method of selecting the final model | **6** |
|  | **27** | Ensembling techniques, if applicable | **N/A** |
| *Evaluation* | **28** | Metrics of model performance | **7** |
|  | **29** | Statistical measures of significance and uncertainty (e.g., confidence intervals) | **8** |
|  | **30** | Robustness or sensitivity analysis | **N/A** |
|  | **31** | Methods for explainability or interpretability (e.g., saliency maps), and how they were validated | **7** |
|  | **32** | Validation or testing on external data | **7** |
| RESULTS |  |  |  |
| *Data* | **33** | Flow of participants or cases, using a diagram to indicate inclusion and exclusion | **8** |
|  | **34** | Demographic and clinical characteristics of cases in each partition | **9** |
| *Model performance* | **35** | Performance metrics for optimal model(s) on all data partitions | **10** |
|  | **36** | Estimates of diagnostic accuracy and their precision (such as 95% confidence intervals) | **11** |
|  | **37** | Failure analysis of incorrectly classified cases | **13** |
| DISCUSSION |  |  |  |
|  | **38** | Study limitations, including potential bias, statistical uncertainty, and generalizability | **15** |
|  | **39** | Implications for practice, including the intended use and/or clinical role | **16** |
| OTHER INFORMATION |  |  |  |
|  | **40** | Registration number and name of registry | **N/A** |
|  | **41** | Where the full study protocol can be accessed | **N/A** |
|  | **42** | Sources of funding and other support; role of funders | **16** |

**Supplementary Table 2.** Acquisition parameters of images used in this analysis.

| **Variable** | **Value** | **N Train** | **N Test** |
| --- | --- | --- | --- |
| Scanner Manufacturer | GE | 0 | 116 |
|  | Philips | 0 | 2 |
|  | Siemens | 324 | 14 |
|  | Toshiba | 0 | 1 |
|  | Unknown | 97 | 9 |
| Reconstruction Kernel | b19f | 142 | 0 |
|  | b30f | 76 | 0 |
|  | b31f | 25 | 1 |
|  | b31s | 31 | 0 |
|  | lung | 0 | 64 |
|  | other | 147 | 50 |
|  | standard | 0 | 27 |
| XR Tube Current (mA) | (0,200] | 228 | 20 |
|  | (200,500] | 95 | 82 |
|  | (500,1000] | 1 | 31 |
| Peak voltage (kVp) | 100 | 0 | 3 |
|  | 120 | 245 | 139 |
|  | 140 | 79 | 0 |
|  | NA | 97 | 0 |
